# State of rare disease management in Southeast Asia

**DOI:** 10.1186/s13023-016-0460-9

**Published:** 2016-08-02

**Authors:** Asrul Akmal Shafie, Nathorn Chaiyakunapruk, Azuwana Supian, Jeremy Lim, Matt Zafra, Mohamed Azmi Ahmad Hassali

**Affiliations:** 1 Social and Administrative Pharmacy, School of Pharmaceutical Sciences, Universiti Sains Malaysia, 11800 Minden, Penang Malaysia; 2 School of Pharmacy, Monash University Malaysia, Jalan Lagoon Selatan, 47500 Bandar Sunway, Selangor Malaysia; 3 Center of Pharmaceutical Outcomes Research, Faculty of Pharmaceutical Sciences, Naresuan University, Phitsanulok, Thailand; 4 School of Pharmacy, University of Wisconsin, Madison, WI USA; 5 School of Population Health, University of Queensland, Brisbane, QLD Australia; 6 Health & Life Sciences, Asia-Pacific, Oliver Wyman, Singapore, Singapore

**Keywords:** Rare disease, Orphan drug, Southeast Asia

## Abstract

**Background:**

Rare diseases, also referred to as orphan diseases, are characterised by their low prevalence with majority of them are chronically debilitating and life threatening. Given the low prevalence and the widely dispersed but very small patient base for each disease, there may often be a disproportion in the availability of treatments and resources to manage patients, spur research and train experts. This is especially true in Southeast Asian countries that are currently in the process of implementing or revising their universal health coverage schemes. This paper aims to examine the status of rare disease management in Southeast Asian countries. It will serve as the basis for a more active discussion on how countries in the region can address an under-recognised rare disease burden and enhance national and regional capacities.

**Methods:**

The study consists of literature reviews and key stakeholders interviews in six focus countries, including the Philippines, Singapore, Malaysia, Indonesia, Vietnam, and Thailand and five countries as best practice, comprising of France, Canada, Australia, Taiwan, and South Korea. Rare disease management initiatives across each country were examined based on the World Health Organization’s framework for action in strengthening health systems.

**Results:**

The results suggest rare disease management remains challenging across Southeast Asia, as many of the focus countries face fundamental issues from basic healthcare systems to funding. Nonetheless, there are substantial improvement opportunities, including leveraging best practices from around the world and organising a multi-stakeholder and regional approach and strategy.

**Conclusions:**

Southeast Asian countries have made significant progress in the management of rare disease, but there remain key areas for substantial development opportunities.

## Background

### The state of rare diseases

Rare diseases are serious and can be life threatening. Even where treatment is available, the disease will likely be a lifetime condition for the patient. While they are characterised by their rarity (less than 1:2000 in Europe, 1:1500 in United States of America, or 1:2500 in Japan), they can collectively affect 1 in 15 persons worldwide [1, 2]. Therefore, while the prevalence of rare diseases in each country is low, total number of people who suffer from rare diseases could be about 400 million worldwide [2]. It is important to note that the prevalence of rare diseases varies based on the type of disease, with some rare diseases affecting 1:100,000 people or less [1]. This rarity heightens the isolation and limited treatment that patients with rare diseases often face.

Despite being “rare,” in Southeast Asia, over 45 million people, or about 9 % of the region’s population, suffer from rare diseases [3]. This number remains contested and it could be even higher. Research and active monitoring of rare diseases is not a priority in Southeast Asia largely due to a lack of resources and inadequate funding. As such, mysterious child deaths in the region may have underlying genetic correlations thus under-reporting the true burden of disease. Out of the eight thousand rare diseases currently characterised in the world, it is likely that several of these rare diseases occur more regularly amongst Southeast Asian populations. For example, Moyamoya disease, which is recognised as a global pathology predominantly affecting children and young adults, is largely present amongst Asian ethnicities [4]. Given the lack of epidemiological data, rare disease landscape and management remain unclear throughout the region. While Southeast Asian countries have made noticeable progress towards universal health care coverage, the focus has been on primary preventive care and acute care [5]. Consequently, funding for rare disease research and treatments remains a central challenge.

### Rarity of diseases limits understanding and appropriate healthcare provision

Due to the limited number of individuals affected by a rare disease, there is lack of experience in managing rare diseases within the local community. Many cases unfortunately result in initial misdiagnosis or failure to diagnose, inadequate treatments, or lack of available treatments [6]. Moreover, without intervention or incentive by the regulatory bodies, pharmaceutical industry may not have the economic motivations to develop treatment for rare diseases given the high cost and small patient base. As such, due to their largely neglected status, pharmaceutical treatments for rare disease t are also call as “orphan drugs.” Because orphan drugs can be expensive and low on public health priority (low prevalence), they were often not reimbursable through public fund. Hence, patients with rare diseases may appear marginalised as most of the funding for their treatment may come from out of pocket, public-private partnerships, charitable organisations, and industry groups (patient assistance programmes) [7]. Moreover, due to the limited understanding and medicinal support, patients may often feel socially and psychologically isolated [6].

### Progress in public awareness and investment opportunities

Public awareness and investment opportunities for rare diseases have increased in recent decades, largely because of work done by patient support groups and The Orphan Drug Act in 1983 in the United States. The Orphan Drug Act creates financial incentives for the research and production of orphan drugs, including tax incentives, patent protections, and clinical research subsidies [8]. Following this enactment, there was a noticeable increase in the number of designated and approved orphan drug by U.S. Food and Drug Administration every decade; from year 1983 (2), 1993 (90), 2003 (238) until 2013 (439) [9]. Continued efforts from the United States spurred the Rare Diseases Act in 2002, establishing an Office of Rare Diseases as a federal entity and increasing national research and investment in the development of diagnostics and treatments for patients with rare diseases [10]. Other countries have followed suite by introducing their own legislation recognising orphan drugs in the past two decades, including Singapore (1991), Japan (1993), Australia (1997), Taiwan (2000), Europe (2002) and South Korea (2003). Despite the progress of orphan drugs and rare disease legislation in some of the countries, disparities remain in the availability of effective treatment for rare diseases globally due to the lack of scientific knowledge and financial incentives. Hence, one of the central suggestions by the World Health Organization in the recent Priority Medicines Report 2013 is to prioritize orphan drugs, by developing new mechanisms to promote the development of basic rare disease research into important treatments [2].

Given the current environment and complexities, the need to understand rare diseases in Southeast Asia is imperative in order to offer suggestions on how the region can better tackle rare diseases and potentially improve healthcare on both a country and regional level.

## Method

### Study setting

In this report, we will review the state of rare disease management in Southeast Asia around several key themes and benchmark them against best practice countries. We have chosen to focus on the six major countries of Southeast Asia: the Philippines, Singapore, Malaysia, Indonesia, Vietnam, and Thailand. These countries were specifically selected on the grounds of economic development, fairly developed national health programmes, and a capacity for regional cooperation and openness. As best practice countries, we have reviewed corresponding rare disease management in five countries: France, Canada, Australia, Taiwan, and South Korea. We have designated these countries as best practice, as key opinion leaders most frequently referenced them and their experience were widely reported in existing literature.

### Data sources

We used multiple sources to obtain relevant information related to rare diseases according to the selected themes. We employed the World Health Organization’s framework for action in strengthening health systems within which to review the status of rare disease management in the focus countries [11]. To supplement the World Health Organization’s evaluation, we reviewed key literature and conducted stakeholder interviews with clinicians, policymakers, advocacy groups, and pharmaceutical experts. The primary and secondary research identified six core themes that were used as criteria for evaluation across the focus geographies and best practice countries.

A comprehensive literature review was performed in June 2014 using electronic databases (PubMed, Google Scholar), published policy documents, and Ministry of Health websites using directed search requests. In addition, supplementary themes were also explored on the management and regulatory requirements of orphan drugs in target benchmark countries. Key opinion leaders were identified through government channels and expert connections. This group included clinicians, policymakers, advocacy groups, and industry leaders. They were consulted using a semi-structured interview approach to improve the understanding of local and regional rare disease issues. The semi-structured interview guide focused on four main issues: i) governance: definition, national strategy or plans, physicians’ involvement, research on rare disease; ii) access: access for the treatment and orphan drugs, support programmes, public awareness; iii) infrastructure: clinical practice guidelines of rare disease, patient registries, neonatal screening programmes, and centre of expertise; and iv) opportunities: training, capacity-building projects, support group, and any strategies suggested.

### Analysis

Information was extracted according to six themes. These themes were used as anchors to identify and assess the national initiatives of rare diseases in the six Southeast Asian geographies as well as the best practice countries. The assessment criteria included:

1. Healthcare system
2. Governance
3. Patient advocacy and rare disease awareness
4. Clinical expertise and patient management
5. Funding
6. New-born screening of rare diseases

Additional insights were captured from the comprehensive literature reviews and interviews of key opinion leaders. The results are presented in Table 1 and discussed in further detail in the following section.

**Table 1:** National initiatives to support rare diseases and access to orphan drugs

	Indicator	Philippines	Singapore	Malaysia	Indonesia	Vietnam	Thailand
Health system	GDP per capita (USD)	2,588	54,007	10,440	3,551	1,755	5,480
	Health expenditure per capita (USD)	119	2,426	410	108	103	215
	Total health expenditure (% of GDP)	4.6	4.7	3.9	3.0	6.6	3.9
	Health coverage (%) [37]	76	100	100	48	55	98
	Out of pocket health expenditure (% of total expenditure on health)	52.0	58.6	35.6	45.3	48.8	13.1
	Life expectancy at birth (years) [40]	69	82	75	70	73	75
	Mortality rate, infant (per 1,000 live births) [40]	22	1.8	7	32	16	11
Governance	Presence of national strategy	Yes	No	In progress	No	No	No
	Definition of rare disease	Yes	Yes	Yes	No	No	No
	Comprehensive rare disease legislation	Yes (2015)	No	No	No	No	No
	Orphan drug act/ legislation	Pending approval	Yes (1991)	No	No	No	No
Awareness	Patient support/advocacy groups	PSOD	RDSS	MLDS, MMA, MRDS	No	NPH RD club	Genetic LSD Foundation
	Patient support group activity	Yes (since 2010)	Yes (since 2013)	Yes (since 2010)	No	Yes (since 2013)	Yes (since 2011)
	Media attention (Based on Factiva search from 2009–2015)	72 articles	48 articles	33 articles	13 articles	26 articles	12 articles
Clinical expertise	Presence of patient registries	Largely institution specific			No	No	Planned, LSD diseases
	Presence of centre of expertise	Yes (1)	Yes (2)	Yes (3)	No	Yes (1)	Yes (2)
	Presence of national guidelines for treatment	No	No	No	No	No	No
	Professional societies to support specialist	No	No	Limited	No	No	No
Funding	Government funding for rare disease treatments	Limited	Limited	Limited	No	No	Limited
	Government funding for rare disease research	No	Yes	No	No	No	No
	Non-government access to rare disease treatment	Some charitable and industry funds	Some charitable and industry funds	Some charitable and industry funds, employer benefits	No	No	Some charitable and industry funds
New born screening	National neonatal screening programs	Yes	Yes	Yes	No	Yes	Yes
	Percentage of new born screened	28 %	100 %	>95 %	<1 %	31 %	>97 %
	Source of payment for the new born screening	OOP/insurance	OOP (40 %)	Gov./ OOP	OOP	Government	

*Abbreviations*: *OOP* Out of Pocket, *Gov* Government, *PSOD* Philippine Society for Orphan Disorders, *RDSS* Rare Disorders Society (Singapore), *MLDA* Malaysia Lysosomal Diseases Association, *MMA* Malaysian Medical Association, *MRDS* Malaysian Rare Disorders Society, *NPH RD* National Pediatric Hospital (rare disease), *LSD* Lysosomal storage diseases

## Results

In general, the six focus countries in Southeast Asia have made healthy progress towards rare disease management. However, the progress differs widely across the themes and region (Table 1). For example, Singapore has a mature health system and several disease awareness initiatives but limited rare disease legislation or funding. On the other hand, the Philippines falls short relative to its peers in terms of health capacity and health expenditure, but it is the first country in the region to introduce a national strategy specifically for rare disease management. In the following, we observe the results for each theme, highlighting countries’ progress and shortcomings.

### Healthcare system

The effectiveness of a country’s healthcare system is considered an important factor for successful development of rare disease management. An effective healthcare system establishes strong foundations for healthcare capacities, capabilities, and budgetary decisions in a country. The six focus geographies differ significantly in their provision of health capabilities and financing, in large part driven by the heterogeneity of economic systems across the region. Singapore has the highest GDP per capita (USD 54,007), health care expenditure (USD 2426), and out-of-pocket expenditure (58.6 %) [11]. This translates to a relatively comprehensive health care coverage for their citizens, low infant mortality rate (1.8 per 1000 live births), and a life expectancy of 82 years. In contrast, Vietnam has the lowest GDP per capita (USD 1775), low health expenditure per capita (USD 103), and a high mortality rate (19.5 per 1000 live births). While economic prosperity may be a strong indicator for a country’s healthcare system, it may be a fallacy that countries must attain economic prosperity before addressing the challenge of rare diseases. Taiwan and South Korea were not top tier income nations before comprehensive support was implemented for rare diseases, a trend apparently replicated in Southeast Asia. Malaysia and the Philippines both have lower GDP per capita (USD 10,440 and USD 2588, respectively) and health care expenditure per capita (USD 119 and USD 410, respectively), however, they either have a rare disease national plan (the Philippines) or are in the process of drafting one (Malaysia).

In addition to the healthcare capacity, the region is also characterised by a range of different healthcare financing schemes with various degree of the potentially catastrophic out-of-pocket payments. Singapore recorded the highest out-of-pocket expenditure (58.6 % of total expenditure on health) for health care while Thailand is the least (13.1 %). However, most of the countries have made significant progress in reducing out-of-pocket health spending. This progress has been analogous with most countries reviewing and strengthening their universal healthcare coverage.

While a country’s financing method and total spending are not by themselves indicators of better health outcomes or achievements in rare disease legislation, it is certainly an important factor that underscores how much attention can be given to rare diseases, assuming other health crises have been sufficiently addressed.

### Governance

Effective governance is another important factor for rare disease management, as it can guide a country’s national strategy and create legislation for rare diseases and orphan drugs. On this criterion, there are marked differences among the six focus countries.

In the region, only the Philippines has a national strategy specifically for rare disease management. The Rare Disease Act of the Philippines was approved in September 2015, covering many key elements including rare diseases, orphan drugs, patients’ care, registry, research and development, information sharing, and screenings [12]. Most importantly, the act categorises patients with rare diseases as ‘persons with disability,’ thus allowing patients to benefit from the statutory benefits provided for in the Republic Act 9442, including discounts on healthcare services and medicines [13].

Other countries are gently advancing their rare disease strategies as well. Malaysia is in the process of developing a national framework for rare disease management. It is currently creating this framework through coordinating national activity, promoting multidisciplinary clinical care of patients and improving on-time diagnosis and treatment. This initiative comes after the government included orphan drugs as one of the elements of the National Medicine Policy in 2012 [14]. Similarly, Singapore adopted the Orphan Drug Act in 1991, expanding drug management by creating a legal framework and promoting the supply of orphan drugs [15]. However, Singapore did not include any financial incentives or additional support for patients, largely due to budgetary concerns.

While transitioning from middle-income status in 2000, Taiwan enacted the Rare Disease and Orphan Act. The act included financial subsidies and exclusive marketing rights for orphan drugs for 10 years [16]. Ten years after implementing the act, the Taiwanese government officially approved 184 rare diseases with 74 orphan drugs and 40 special nutrients. The number of rare disease patients had also increased from about 4200 people (2004) to more than 6000 people (2011). Moreover, all Taiwanese rare disease sufferers are eligible to get at least 70 % reimbursement on orphan drugs, whereas low-income citizens can receive 100 % reimbursement [17]. This made Taiwan as one of the best practice model of comprehensive rare disease management governance.

Effective governance is critical to the development of rare disease management; however, it is important to underline that the government is not the only key player in pushing forward a strategy for rare disease management. In the Philippines, the Rare Disease Act approved in September 2015 has strong backing from patient support groups who took initiatives to map out the national strategy [18]. Additionally, in Taiwan, the advocacy groups also strongly support rare disease patients and the country legislation.

### Patient advocacy and rare disease awareness

Awareness is a critical factor in rare disease management as it leads to greater public and private involvement. Over the past 5 years, public awareness of rare diseases has increased in Southeast Asia. Most of the focus countries (except Indonesia) have their own patient support groups such as the Philippine Society for Orphan Disorders (the Philippines), Rare Disorders Society (Singapore), Malaysian Rare Disorder Society (Malaysia), and Genetic LSD Foundation (Thailand). Beside information from their doctors, the patients can search and get more information from these societies in their country. Like other countries, these groups involved in many rare disease activities. They hosting an awareness campaign and local rare disease conference, produce traditional and new media such as television programmes, publications and blogs as well as social media (Facebook). Every year they organise rare disease day, charity run, fundraising and donation. To sustain the networking with others, they also participate especially in rare disease meetings and conferences locally as well as around the world [19–21]. Their efforts have resulted in strong media attention in the Philippines, Singapore, and Malaysia, based on hit rates more than 30 in the Factiva search conducted as part of the study.

In particular, the Philippines reports strong support and activity from patient and advocacy groups. The primary group, Philippine Society for Orphan Disorders, had drafted the national rare disease strategy for the Philippines and is a core proponent of the recently approved legislation, the Rare Disease Act of the Philippines (2015) [18].

Singapore may also now increase public awareness, as it hosted the first Rare Disease Asia Conference in February 2015. The conference brought together 25 patient support groups from 13 countries. Moreover, it has established the *Rainbow Across Borders* patient support organisation that acts as a regional umbrella alliance for rare diseases [22]. This organisation promotes regional collaboration and networking among patient support organisations from countries within the Asia-Pacific region. Its objectives are to empower patient support organisations through appropriate programmes, services and training, while facilitating learning, and experience exchange among the affiliates. The organisation also focuses on developing the rare disease registry and directory across Asia.

Overall, the existence of patient advocacy across Southeast Asia is a positive sign and highlights the severity and pressing need to tackle the challenge of rare disease management. Southeast Asian groups could emulate the practice and model of other patient advocacy group in reference country like Australia. Over 200 rare disease patient organisations are unified under Australia’s National Alliance for rare diseases, named Rare Voices Australia (RVA) [23]. RVA is established in 2012, which provides a strong common voice to promote for health policy and a healthcare system that works for those with rare diseases. It also work with a vast range of stakeholders including Federal and State governments, researchers, health professionals and industry to promote and advocate for equity within the health care system.

### Clinical expertise and patient management

Clinical expertise and patient management of rare diseases varies widely across the region, need to be improved. For example, patient registries are critical in the management of rare diseases; however, there are no national rare disease patient registries in the region. Rather, most countries have separate lists or databases in individual institutions or treating facilities. This leads to potential risks such as redundant entries of patients, producing incomplete data on the prevalence of rare diseases. No country has proposed improvements, except for Thailand who plans to create a nationwide database of lysosomal storage diseases.

In addition, there is a lack of clinical rare disease expertise across the region. There are only a limited number of genetic specialists and only a few institutional centres that offer specific services to treat rare diseases. For example, in Thailand, there are only 22 geneticists available to serve the whole population of 67 million people, with most of them located in major cities. Moreover, there is no professional society focused on rare diseases to support those specialists, collecting and sharing local experiences. As a result, genetic specialists often do not have the critical mass to influence the direction of funding and availability of specialised care.

In 2014, Care for Rare, a pan-Canadian research team has developed an online database named Phenome Central. It is created as a hub to bring doctors and scientists working on rare diseases together [24]. This online database is a great initiative to help clinicians and rare disease scientists all over the world to share their case records and information in the database, which can be used by rare disease clinicians in SEA region.

### Funding

Funding in rare diseases is central to the discussion of rare disease management and reaches across key stakeholders, including patients, the government, and the private sector. Due to high cost of treatment for relatively few individuals, there are often worries that coverage of rare diseases will undermine the financial sustainability of private investment as well as a country’s universal healthcare coverage. Government funding for rare disease treatments is limited and ad hoc across Southeast Asia. Only a small number of patients have access to treatment. For example, the Malaysian government subsidises only certain enzyme replacement therapies and treatments such as *alglucosidase alfa* and *elaprase*. Moreover, this limited funding is only available for selected patients meeting a number of selective criteria for eligibility and only through hospitals run by the Ministry of Health [25]. Nonetheless, Malaysia is one of the few countries offering some form of public funding for rare diseases. In other countries, the large majority of rare disease financing is through industry subsidisation, employer benefits, charitable work or out-of-pocket payment. In Singapore, only a few rare disease patients receive subsidies from the industry, hospital funds, or Medifund, the national means-tested fund for indigent patients.

Another issue in the region is there is no systematic mechanism for rare disease funding and rather, a sporadic and inconsistent use of cost assessment frameworks to guide funding decisions. According to National Institute for Health and Care Excellence guidelines, a medical treatment is considered cost effective if the incremental cost-effectiveness ratio is below GBP 30,000 per quality-adjusted life year gained [26]. By using this method, most orphan drugs are unlikely to be cost-effective. For example, treatment for infantile Pompe disease has an incremental cost-effectiveness ratio estimated at GBP 1,000,000, which is more than 30 times higher than the threshold [27]. For this reason, a fit-for-purpose process is needed which may consider other factors in addition to its cost-effectiveness. In Thailand, the ceiling threshold of incremental cost-effectiveness ratio is below THB160,000 per quality-adjusted life year. Yet, for Type 1 Gaucher disease, there were budget impacts and equity issues that rendered it a positive economic choice, despite the unattractive ICER (THB6,300,000 per quality-adjusted life year or 50 times greater than the cost-effectiveness threshold) [28]. This success is expected to heighten awareness of funding authorities throughout the SEA region.

The message is that by concentrating on costs and its cost-effectiveness only, we will miss the broader picture behind the rare diseases management and equitable access to healthcare. Thailand’s example may provide opportunities for other orphan drugs to be reimbursed as well. Furthermore, only small percentage of total health expenditure are allocated for rare disease treatment compared to other diseases; for example Europe and United States spends about 3.3 and 7.7 % respectively [29, 30].

### New-born screening of rare diseases

The onset of rare diseases typically occurs in children for 50 % of rare diseases [6]. It is therefore a common procedure in most health facilities to screen newborn babies for selected congenital illnesses amenable to early intervention and treatment. Early intervention helps to prevent serious problems, as some medical conditions are indiscernible. Each of the focus countries, with the exception of Indonesia, has an established newborn screening policy with various degrees of funding, participation and implementation. In addition, the lack of access to reliable neonatal testing facilities and a limited number of specialists contribute to the delay of diagnosis. Many of the rare disease tests also must be sent abroad for analysis, which adds to the total cost of treatment.

In the Philippines, a new-born screening centre was established in 1997 to serve as the central laboratory that providing advanced tests for the National Comprehensive New-born Screening System [31]. Even though the National Neonatal Screening Plan is in place, the approximately USD 12 out of pocket cost of neonatal screening is still a burden for many poor families as the annual income of Filipinos was less than USD 5000 per annum or around USD 13.7 per day in 2012 [32, 33].

New-born screening began in Malaysia in 1980 with cord-blood screening for glucose-6-phosphate dehydrogenase deficiency [34]. Many of the newborn tests however are not mandatory including amino acid metabolism, fatty acid oxidation, and organic acid metabolism disorders. Malaysia expanded newborn screening which included inborn errors of metabolism. Nevertheless, the expanded tests are only available mainly in two centres (Institute for Medical Research and Centre for Advanced Analytical Toxicology Services) at the request by public hospitals and some private hospitals [34]. Additionally, Malaysia still faces a dearth of information and resources, which reduces the uptake of the newborn screening policy; these include a limited number of specialists and the perception among parents that new-born screening is optional.

Singapore provides more than 25 metabolic related screening tests for newborn babies under the National Expanded New-born Screening Programme. The programme offers screening of inborn errors of metabolism to all babies born in Singapore [35]. Nevertheless, more complex tests including lysosomal storage diseases are still sent to Japan and Taiwan as they have the only screening facilities for these types of disorders in Asia [36].

## Discussion

From the broad overview of rare disease initiatives across the focus countries in SEA, we have developed the following observations and recommendations:

1. Rare disease management remains immensely challenging across the region, as costs remain high and resources are limited.
2. Countries in SEA are largely disparate in the advancement of rare disease management.
3. There are substantial improvement opportunities across all aspects.
4. It is important for each focus country to adopt and adapt best practices from around the world.
5. There is potential to organise a multi-stakeholder and multi-country approach and strategy to manage rare diseases efficiently.

The observations and recommendations highlight the opportunities for the region to leverage best practices within SEA and worldwide, in order to improve rare disease management on a country and regional level. However, the six focus geographies face huge challenges and thus, the effort should be on making incremental steps towards a regional model to manage rare diseases efficiently.

### Rare disease management remains immensely challenging across the region, as costs remain high and resources are limited

There are major barriers toward effective rare disease management worldwide. The challenging nature of rare diseases, most notably their low incidence rate, renders management difficult as the average annual cost per person works inverse to prevalence. Moreover, the increasing number of orphan drugs and budget impact continue to concern decision makers. As such, governments worldwide face a problem in prioritising funding for orphan drugs. In particular to Southeast Asia, over 45 million people in the region are estimated to suffer from rare diseases [3]. However, Southeast Asia also shoulders other health challenges (e.g. cardiovascular disease) that are more common, and therefore more publicised. With fewer resources than those in many other areas of the world, the region has historically focused its health policy on ensuring primary and preventive care, acute care, and management of disease with high epidemiologic burden. The concentration on broadening access in such areas understandably leaves little room for funding, let alone discussions, on rare diseases. Despite limited prioritisation, horizontal and vertical equity arguments from various stakeholders, including patient advocacy groups, have elevated the need to tackle rare diseases across the region. We can accordingly observe that Southeast Asian countries consider rare diseases initiatives as in scope and salient, although current efforts are fragmented and dispersed across the region.

### Countries in southeast Asia are largely disparate in the advancement of rare disease management

While there are positive advancements throughout Southeast Asia, barriers remain and health systems differ widely across borders. Across the region, growing awareness of rare diseases is grounded on patient support and advocacy groups. In all of the focus countries, excluding Indonesia, there is at least one active patient support group, along with the regional alliance for rare diseases, *Rainbow Across Borders*. Despite this, there remain persistent limitations, including inadequate funding, lack of clinical expertise, and patient management.

With regard to the health system and governance, the six focus countries differ remarkably. Singapore spends 20 times more on health per capita than Vietnam, Indonesia, and the Philippines [33]. In addition, Singapore covers 100 % of its population, while Indonesia, Vietnam, and the Philippines have significantly lower health coverages (48, 55, and 76 %, respectively) [37]. Despite this, the Philippines has made tremendous progress in formulating their rare disease national strategy, including a national definition of rare disease and comprehensive rare disease legislation. It included management of persons with rare diseases, designation of rare disease and orphan drug, role of agencies and incentives for rare diseases [12]. While Singapore Orphan Drug Act has gazetted only management and supply of orphan drug information [15]. There is no formal governance structure for rare disease in Thailand, Vietnam, and Indonesia. Except for Indonesia, the other focus countries have an established national neonatal screening programme with various degree of participation. While Singapore and Thailand screen more than 97 and 100 % of their new-born babies respectively, the Philippines and Vietnam only screen 28 and 31 %, respectively [36].

### There are substantial improvement opportunities across all aspects

Southeast Asia faces significant gaps across the rare disease management themes. With regard to the overarching health system, resources, outcomes, and funding differ widely, with most of the region prioritising chronic and communicable diseases. Given this, there may be muted political will and commitment to support rare diseases. While strong patient advocacy groups exist, the lack of patient registries with standardised information renders understanding the situation in the region difficult. In addition, there is a lack of infrastructure to support medical care intervention and limited standardisation of guidelines for treatment. Finally, the region also sees inequitable funding for treatments, with a strong perception that coverage of rare diseases will undermine the financial sustainability of current basic universal coverage systems.

Given these complexities, the focus geographies could substantially improve and develop stronger rare disease management platforms consisting of advocacy and awareness promotion, partnerships and sharing within and between countries, support for rare disease research, and holistic funding models. This could be developed with the combined effort of patients, families, and physicians, working with advocacy groups to help increase public and political awareness. In addition, countries could share available information for clinicians, researchers, and patients. Finally, it is feasible and crucial for the region to adopt and adapt best practices from around the world, and develop partnerships and regional cooperation across Southeast Asia and internationally.

### It is important for each focus country to adopt and adapt best practices from around the world

**Fig. 1:**
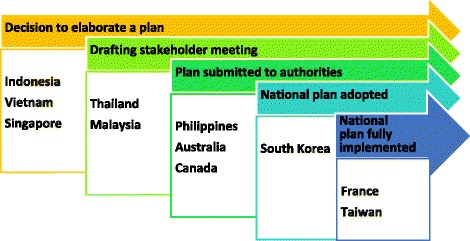
Rare disease journey-Progress of SEA and benchmark countries

To develop a good national rare disease plan is not an easy task for many countries. Some other countries typically take a long journey from decision to elaborate a national rare disease plan to full or even partial implementation. Even many benchmark countries like Australia and Canada also have yet to reach the end of path (Fig. 1). Therefore, cooperation with regional and international is essential in rare disease management. SEA countries can adopt and adapt the best established practices and approaches from benchmark countries. A critical fallacy to be corrected is that countries must attain economic prosperity before focusing on rare diseases. For examples, Taiwan and South Korea were not top tier income nations before comprehensive support to rare disease was implemented.

Again, Taiwan is a good example of what is possible. The Rare Disease and Orphan Drug Act 2000, has enshrined special pricing rules and regulations. After 10 year of its implementation, the Taiwanese government officially approved 184 rare diseases with 74 orphan drugs and 40 special nutrients [16]. Number of rare disease patients has also increased from about 4200 people (2004) to more than 6000 people (2011). Under this act, all Taiwanese rare disease sufferers are eligible to get at least 70 % reimbursement. Whereas for low-income citizens, they can receive 100 % reimbursement of actual orphan drugs and nutritional supplements expenses [38]. SEA countries can adopt and adapt the Rare Disease Control and Orphan Drug Act of Taiwan which control their listing of covered orphan drugs and implement medical subsidies for patients with rare diseases [39].

### There is potential to organise a multi-stakeholder and multi-country approach and strategy to manage rare diseases efficiently

**Fig. 2:**
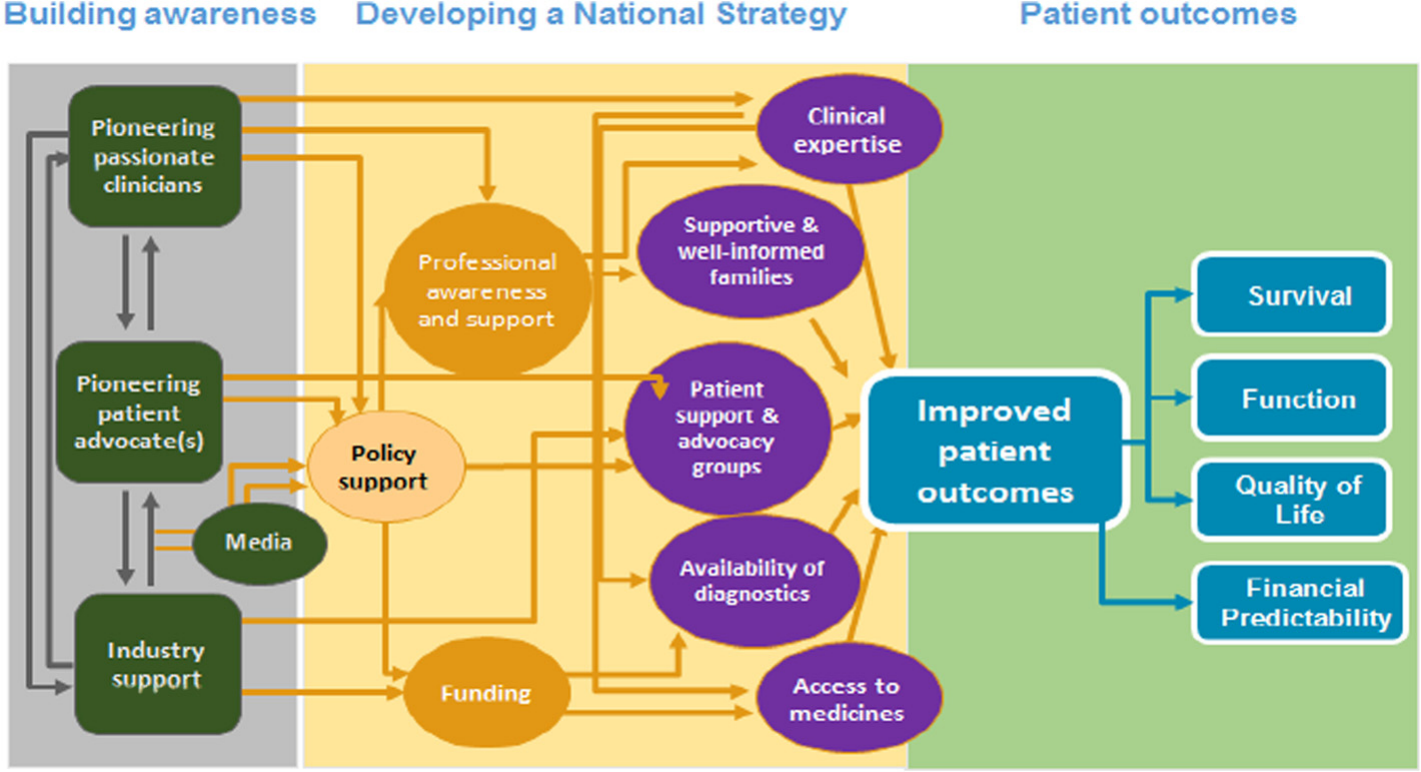
Rare disease framework-identifying stakeholders, support and measures of success

Management of rare disease should highlight the necessity comparable with other common diseases in term of treating rare disease sufferers, sharing information of registries, competencies, advocacy and awareness of the disease. Improvement of all these issues should be driven by all parties especially the government, economic realities, political support, clinical expertise, industry as well as patient activism towards better patient outcomes (Fig. 2).

There are possibilities for SEA to improve cross border cooperation in rare disease support for instance free flow of goods, services, investment or capital and skilled labour. With all six initiatives, it can develop a regional model for rare disease to support rare disease registry, genetic database, training, education, awareness besides saving time and costs. Few incremental steps and focus actions are recommended and should be taken by Association of SEA Nations (ASEAN) towards comprehensive rare disease treatment and funding (Table 2).

**Table 2:** Proposed focus actions for Association of SEA Nations (ASEAN)

1.	Promote equity and increase focus on: a) develop a Rare Disease Plan, b) establish ASEAN network on rare disease c) continuous patient advocacy in ASEAN region;
2.	Consistency of clinical services by: a) developed standard treatment guidelines, b) identify and invest in rare disease Centre of Excellence, c) establish the national registry for rare disease patients d) regional sourcing of orphan drugs for greater purchasing power;
3.	Efficient research consortium with: a) enough fund for research b) genetic database to collect information from these unfortunate patients

Nevertheless, the most important is the partnerships with government, policymakers, clinician, patient support groups and industry within and even between countries. Hopefully, these suggestions can be adopted and address some of the problems or issues in managing rare diseases and orphan drugs access.

## Conclusion

Generally, Southeast Asian countries have made significant progress in the management of rare disease, but there remain key areas for substantial development opportunities. We offer the following short and medium-term suggestions with the underpinning belief that a country’s progress of rare disease management is in parallel with the development of its universal healthcare system and infrastructure.

In the short term, focus countries should understand and leverage best practices and established approaches from around the world. In rare disease management, countries typically take a similar journey and thus there is no need to reinvent the wheel. More importantly, while best practice countries are relatively wealthy, many of them show it is not a prerequisite to building out rare disease management. Additionally, focus countries should support and leverage patient support and advocacy groups. Highlighting the case for rare disease sufferers will help with sensitivity and understanding across different stakeholders. It will also move the needle forward on a comprehensive rare disease national strategy and legislation, including efforts for public and private funding.

In the medium term, it is crucial for the region turn to information sharing, partnerships, and ultimately a regional model. Each country might not have enough resources or even patients to tackle rare disease management. However, scale exists in aggregate to develop initiatives and share expertise, medicines, and technology. One-step could be to establish an ASEAN-wide rare disease network made up of clinicians, healthcare policymakers and advocacy groups. In addition, similar to the establishment of *Rainbow Across Borders* organisation, the region should continue region-wide patient advocacy for rare disease patients and increase awareness. Another incremental step should be centred on clinical services for consistency. Countries on a national and regional level should develop standardised treatment guidelines, identify and invest in centres of excellence, establish compatible national patient registries, enable information sharing as well as improve regional sourcing of drugs for greater purchasing power. Finally, research consortiums could be developed for greater efficiency. This should include increased funding for research into rare diseases and a creation of a genetic database to collect information from rare disease sufferers.

Ultimately, if the focus countries work together to manage rare diseases, the region will see an improvement of patient outcomes through better funding and community support, leading to a better understanding of rare disease patient needs and how to improve their daily experiences. This in turn will also improve the overall healthcare landscape of Southeast Asia.

### Limitations of the study

Public information and industry and statistical data are from sources we deem to be reliable based on current data and historical trends. Information from communications and interviews are assumed reliable. Any such predictions are subject to inherent risks and uncertainties.

## Abbreviations

AEC, ASEAN Economic Community; ASEAN, Association of SEA Nations; CAATS, Centre for Advanced Analytical Toxicology Services; ERT, Enzyme replacement therapy; FDA, U.S. Food and Drug Administration; GDP, Gross Domestic Product; GBP, British Pound; ICD, International Classification of Diseases; ICER, Incremental cost-effectiveness ratio; IEM, Inborn errors of metabolism; IMR, Institute for Medical Research; NBS, New born screening; QALY, Quality-adjusted life-year; SEA, Southeast Asia; THB, Thai Baht; UHC, Universal Health Coverage.
